# Needle Trajectory Influences Foraminal Contrast Distribution and Pain Reduction Following Paramedian Cervical Interlaminar Epidural Steroid Injection: A Retrospective Study

**DOI:** 10.3390/medicina62050976

**Published:** 2026-05-17

**Authors:** Seounghun Lee, Jiho Park, Juyeon Kim, Yeojung Kim

**Affiliations:** 1 Department of Anesthesiology and Pain Medicine, Chungnam National University Sejong Hospital, Sejong 30099, Republic of Korea; 2 Department of Anesthesiology and Pain Medicine, Chungnam National University College of Medicine, Daejeon 35015, Republic of Korea; 3 Department of Anesthesiology and Pain Medicine, Chungnam National University Hospital, Daejeon 35015, Republic of Korea

**Keywords:** cervical epidural steroid injection, paramedian approach, needle trajectory, contrast spread, foraminal distribution, periradicular spread, radicular pain, fluoroscopy

## Abstract

*Background and Objectives*: Paramedian cervical interlaminar epidural steroid injection (CESI) is commonly used for cervical radicular pain and is considered safer than the transforaminal approach. However, its clinical effectiveness may be influenced by contrast distribution patterns, although these may not fully reflect actual drug delivery. This study aimed to evaluate the association between needle trajectory, foraminal or periradicular contrast distribution patterns, and short-term pain reduction following paramedian cervical interlaminar CESI. *Materials and Methods*: This single-center retrospective study included 109 patients who underwent paramedian cervical interlaminar CESI. Needle trajectory was classified as inward or outward. Contrast distribution was graded based on anteroposterior (AP) spread patterns. Pain intensity was assessed using a numeric rating scale (NRS) at baseline and 2 weeks after the procedure. Group comparisons were performed using Welch’s *t*-test and chi-square or Fisher’s exact test, as appropriate. Effect sizes were calculated using Cohen’s d and η^2^. Multivariable linear regression analysis was performed adjusting for age, sex, baseline NRS, and target level. *Results*: The outward trajectory group demonstrated a significantly higher proportion of Grade 2 contrast spread compared to the inward group (69.8% vs. 8.9%, *p* < 0.001). Higher AP contrast spread grades were associated with greater pain reduction (β = 0.83, 95% CI: 0.44–1.22, *p* < 0.001; η^2^ = 0.14). In addition, patients in the outward trajectory group showed greater NRS reduction than those in the inward group (2.96 vs. 1.71, mean difference: 1.25, 95% CI: 0.74–1.76, *p* < 0.001; Cohen’s d = 0.96). In multivariable analysis, needle trajectory remained significantly associated with pain reduction, whereas AP contrast spread grade was not independently associated. *Conclusions*: Needle trajectory was associated with contrast distribution patterns and short-term pain reduction following paramedian cervical interlaminar CESI. An outward-directed trajectory was associated with greater foraminal or periradicular contrast spread and greater pain reduction. These findings suggest that needle trajectory may represent a clinically relevant procedural factor influencing clinical outcomes.

## 1. Introduction

Cervical epidural steroid injection (CESI) is widely used for the treatment of cervical radicular pain [1,2,3,4]. The primary goal of CESI is to deliver anti-inflammatory medication into the epidural space to reduce inflammation of the affected nerve root and alleviate pain [5,6,7].

Cervical transforaminal epidural steroid injection (TFESI) has traditionally been considered a targeted technique that allows direct delivery of medication to the affected nerve root [8,9]. However, increasing concerns regarding serious complications, including spinal cord infarction and catastrophic neurologic injury, have led to a decline in its use in the cervical region [10,11,12,13]. Consequently, interlaminar CESI has gained wider acceptance due to its more favorable safety profile.

Despite these advantages, interlaminar CESI has limitations in achieving targeted drug delivery. Previous studies have shown that contrast spread within the cervical epidural space is highly variable and may not consistently reach the neural foramen or periradicular region [14,15,16,17]. This variability has been suggested as a potential explanation for inconsistent clinical outcomes [15,18]. However, contrast distribution does not necessarily reflect actual drug delivery or predict analgesic efficacy [16,17].

To improve target-specific delivery, the paramedian interlaminar approach has been proposed as an alternative to the conventional midline approach, as it may facilitate more lateral distribution of injectate toward the neural foramen [14,19,20]. However, variability in contrast spread patterns persists even with this approach, suggesting that additional technical factors may influence injectate distribution.

One such factor may be needle trajectory. The direction of needle advancement—whether inward or outward—may affect contrast distribution, particularly foraminal or periradicular spread. However, the impact of needle trajectory on contrast distribution patterns and its clinical relevance has not been clearly established.

We hypothesized that an outward-directed needle trajectory would result in greater foraminal or periradicular contrast distribution and improved short-term pain outcomes. Therefore, the aim of this study was to evaluate the association between needle trajectory, contrast spread patterns, and short-term pain reduction in patients undergoing paramedian cervical interlaminar epidural steroid injection.

## 2. Materials and Methods

### 2.1. Study Design and Participants

This single-center retrospective observational study included patients who underwent paramedian cervical interlaminar epidural steroid injection (CESI) between April 2024 and September 2025 at Chungnam National University Sejong Hospital.

The study was conducted in accordance with the Declaration of Helsinki and was approved by the Institutional Review Board of Chungnam National University Sejong Hospital (IRB No. CNUSH 2026-01-005). The requirement for written informed consent was waived due to the retrospective design and use of anonymized data. The retrospective design may limit causal inference and introduce the potential for selection bias.

### 2.2. Patient Selection and Clinical Evaluation

Eligible patients were adults (≥19 years) diagnosed with cervical radiculopathy presenting with upper extremity radiating pain associated with cervical disc herniation, foraminal stenosis, or degenerative cervical spine disease.

The inclusion criteria were as follows:

1. Patients who underwent paramedian cervical interlaminar CESI at our institution;
2. Availability of fluoroscopic images, including anteroposterior (AP) and contralateral oblique (CLO) views;
3. Availability of post-contrast AP images for evaluation of contrast spread;
4. Documented baseline and 2-week follow-up pain scores;
5. Complete clinical and imaging data available in the electronic medical records.

The exclusion criteria were as follows:

1. Prior cervical spine surgery (e.g., fusion or disc replacement);
2. Non-degenerative causes of cervical pain (e.g., trauma, infection, tumor, or inflammatory disease);
3. Intravascular contrast uptake during the procedure;
4. Poor-quality fluoroscopic images precluding evaluation;
5. Additional interventional procedures or surgery within 2 weeks after CESI;
6. Missing pain score data.

In addition, patients with a needle tip located in the medial half of the interlaminar space on anteroposterior fluoroscopic view were excluded, as such positioning was not consistent with the predefined paramedian approach.

**Figure 1:**
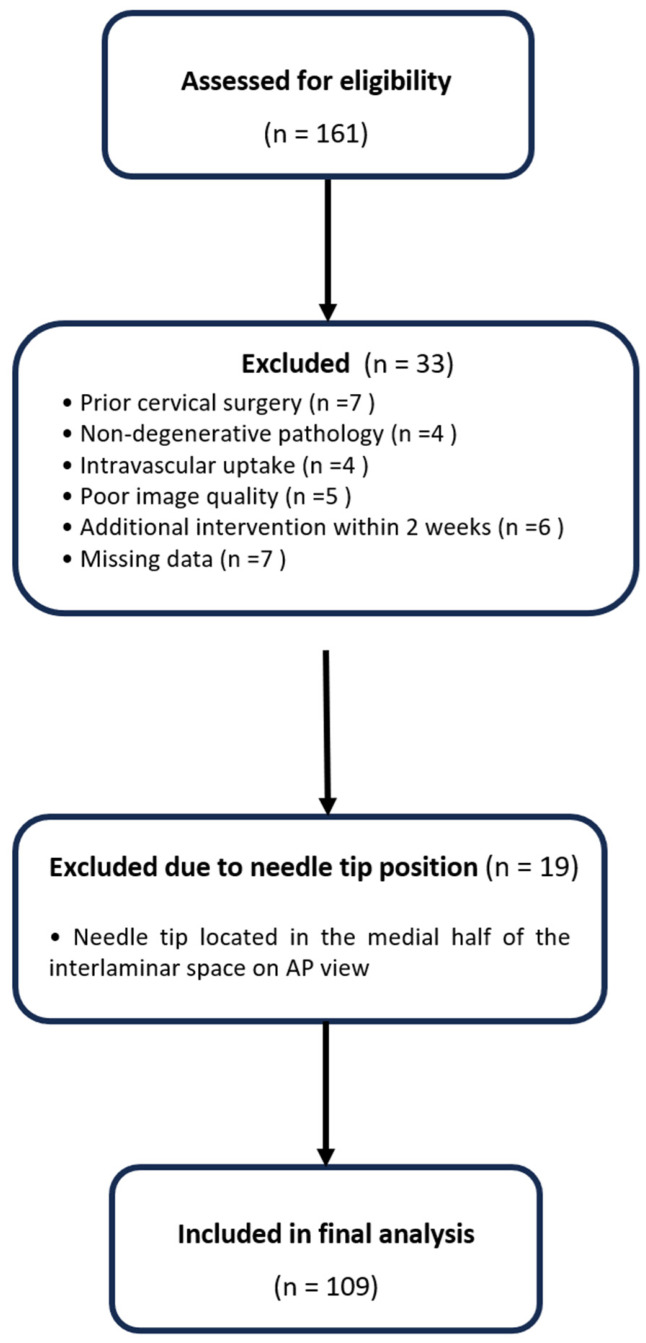
Flow diagram of patient selection. A total of 161 patients were assessed for eligibility. After excluding 33 patients based on predefined criteria and 19 patients due to needle tip location in the medial half of the interlaminar space on anteroposterior view, 109 patients were included in the final analysis.

Pain intensity was assessed using a numeric rating scale (NRS; 0–10). Pain reduction was defined as the absolute change in NRS score from baseline to 2 weeks. The short follow-up period limits assessment of long-term treatment durability and sustained clinical outcomes. A flow diagram illustrating patient selection is shown in Figure 1.

### 2.3. Procedure Technique

All procedures were performed under fluoroscopic guidance by a single experienced pain physician. Patients were placed in the prone position with slight neck flexion to optimize access to the interlaminar space. Procedures were conducted using a C-arm fluoroscopy system (Arcadis Varic, Siemens Healthineers, Erlangen, Germany).

After standard sterile preparation, local anesthesia was achieved with 1% lidocaine (Huons Co., Ltd., Seongnam, Republic of Korea). A 20-gauge Tuohy needle (B. Braun Melsungen AG, Melsungen, Germany) was inserted using a paramedian approach and advanced toward the epidural space using a loss-of-resistance (LOR) technique with saline. Real-time anteroposterior (AP) and 50-degree contralateral oblique (CLO) fluoroscopic views were used to confirm appropriate needle depth and trajectory during the procedure [21].

Correct epidural placement was confirmed by injection of 1–2 mL of contrast medium (iopamidol 300 mg/mL; Iopamiro 300, Bracco Imaging S.p.A., Milan, Italy) under fluoroscopy. Although the volume of contrast medium was not strictly standardized, injection was performed under real-time fluoroscopic guidance and was adjusted based on visualization of epidural spread. The injection was terminated once adequate epidural distribution was achieved, reflecting routine clinical practice. Because of the retrospective nature of the study, the exact injected contrast volume was not consistently recorded in all procedural reports; however, the intended contrast volume was generally limited to 1–2 mL according to institutional procedural practice.

Needle trajectory was classified based on the overall direction of advancement relative to the midline on AP fluoroscopic view, reflecting real-world procedural practice:

- Inward trajectory: needle directed medially toward the midline;
- Outward trajectory: needle directed laterally toward the neural foramen.

After confirmation of epidural placement, a total of 4 mL of injectate (3 mL of 0.2% ropivacaine [Mitsubishi Tanabe Pharma Korea Co., Ltd., Seoul, Republic of Korea] and 1 mL of dexamethasone [5 mg/mL; Yuhan Corporation, Seoul, Republic of Korea]) was administered slowly over approximately 30 s. Procedures performed by a single operator ensured technical consistency but may limit generalizability to other operators or clinical settings. All patients were monitored for 30 min after the procedure.

### 2.4. Imaging Analysis and Outcome Measures

#### 2.4.1. Contrast Spread Evaluation

Fluoroscopic images obtained immediately after contrast injection were reviewed to assess contrast distribution.

**Figure 2:**
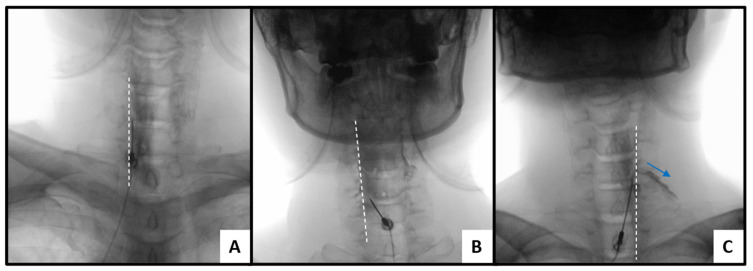
Representative fluoroscopic images demonstrating anteroposterior (AP) contrast spread patterns. (**A**) AP grade 0: contrast confined medial to the pedicle medial margin (dashed line), without extension toward the foraminal region. (**B**) AP grade 1: contrast reaches or slightly crosses the medial margin of the pedicle (dashed line), suggesting limited lateral epidural spread. (**C**) AP grade 2: contrast clearly extends beyond the pedicle into the foraminal or periradicular region (arrow), indicating more extensive lateral distribution.

AP contrast spread was graded based on the relationship between contrast distribution and the medial margin of the pedicle as follows (Figure 2):

- AP grade 0: contrast confined medial to the pedicle medial margin, without extension toward the foraminal region.
- AP grade 1: contrast reaching or slightly crossing the pedicle medial margin, suggesting limited lateral epidural spread.
- AP grade 2: contrast clearly extending beyond the pedicle into the foraminal or periradicular region.

The medial margin of the pedicle was selected as a clinically relevant anatomical landmark to distinguish central from foraminal/periradicular contrast distribution, as it corresponds to the transition toward the neural foramen on fluoroscopic imaging [16,22].

**Figure 3:**
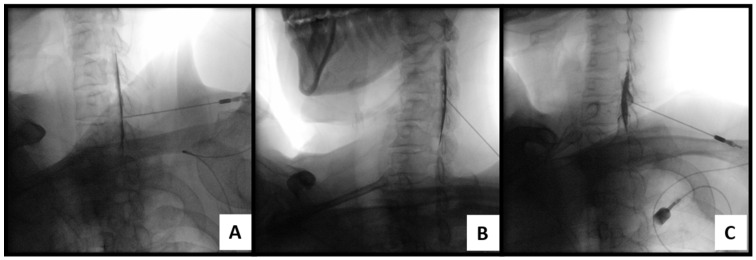
Representative fluoroscopic images demonstrating ventral epidural contrast spread patterns on contralateral oblique (CLO) view. (**A**) Ventral grade 0: contrast confined to the posterior epidural space without anterior extension beyond the posterior vertebral body line. (**B**) Ventral grade 1: faint or equivocal extension of contrast along the posterior vertebral body margin, suggesting possible ventral epidural spread. (**C**) Ventral grade 2: definite and continuous anterior extension of contrast along the posterior vertebral body margin, consistent with ventral epidural spread.

Ventral epidural spread was assessed using contralateral oblique (CLO) fluoroscopic views and graded as follows [22] (Figure 3):

- Ventral grade 0: contrast confined to the posterior epidural space, without anterior extension beyond the posterior vertebral body line.
- Ventral grade 1: faint or equivocal extension along the posterior vertebral body margin, suggesting possible ventral spread.
- Ventral grade 2: definite and continuous anterior extension of contrast along the posterior vertebral body margin, consistent with ventral epidural spread.

Ventral spread was analyzed as an exploratory variable to assess its potential additional contribution to contrast distribution patterns and was not used as a primary outcome variable.

Contrast spread classification was based on a semi-quantitative assessment, which may introduce observer-related variability despite high inter-rater agreement.

Contrast spread patterns and needle trajectory were independently reviewed by two experienced pain physicians who were blinded to clinical outcomes. In cases of disagreement, a consensus was reached through joint review. Inter-rater agreement for AP grade classification was excellent (Cohen’s κ = 0.92), indicating high consistency between reviewers.

The grading system was based on previously reported fluoroscopic approaches that evaluate contrast distribution relative to anatomical landmarks [18,22].

#### 2.4.2. Clinical Outcome Assessment

Pain intensity was assessed using a numeric rating scale (NRS; 0–10), where 0 indicates no pain and 10 indicates the worst imaginable pain, at baseline and at 2 weeks after the procedure. Pain reduction was defined as the absolute change in NRS score from baseline to 2 weeks.

### 2.5. Statistical Analysis

All statistical analyses were performed using R software (version 4.5.2; R Foundation for Statistical Computing, Vienna, Austria). Continuous variables are presented as mean ± standard deviation, and categorical variables as counts and percentages. Continuous variables were compared using Welch’s two-sample *t*-test, and categorical variables using the chi-square test or Fisher’s exact test, as appropriate.

Associations between needle trajectory and contrast spread (AP grade) were evaluated using the chi-square test. Linear regression analysis was performed to assess the association between AP contrast spread grade and pain reduction.

To account for potential confounding factors, multivariable linear regression analysis was additionally performed as an exploratory analysis, adjusting for age, sex, baseline NRS, and target level.

Effect size measures were calculated to quantify the magnitude of observed differences. Cohen’s d was used for group comparisons, and η^2^ was used to estimate effect size for variance-based analyses. Effect size interpretation was based on previously reported benchmarks for pain research, with values of approximately 0.10, 0.30, and 0.70 corresponding to small, medium, and large effects, respectively [23].

All statistical tests were two-sided, and a *p*-value < 0.05 was considered statistically significant.

## 3. Results

### 3.1. Patient Characteristics

A total of 109 patients were included in the final analysis, with 56 patients in the inward trajectory group and 53 in the outward trajectory group.

Baseline demographic and clinical characteristics are summarized in Table 1. There were no significant differences between the two groups in age, sex distribution, target level, injection side, or baseline NRS scores. However, the outward trajectory group demonstrated significantly lower 2-week NRS scores and greater pain reduction compared to the inward trajectory group. The difference in pain reduction showed a large effect size (Cohen’s d = 0.96).

### 3.2. Association Between Needle Trajectory and Contrast Spread

**Figure 4:**
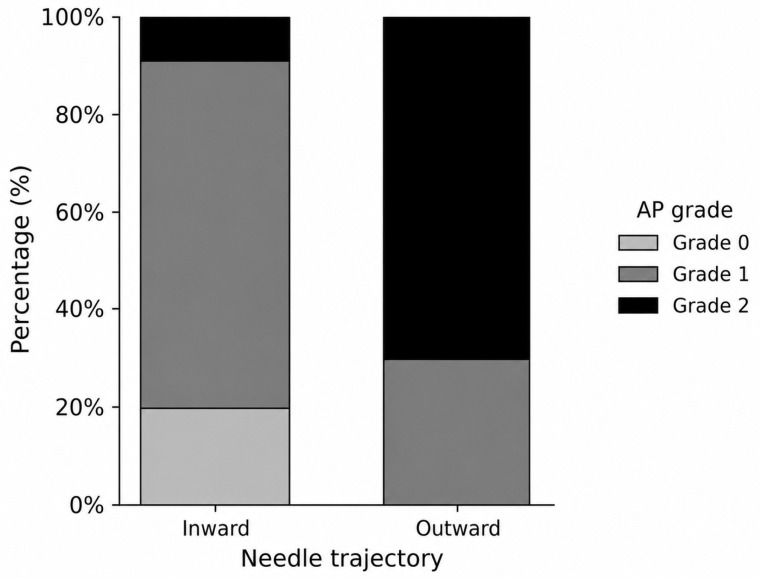
Distribution of AP contrast spread grades according to needle trajectory.

The distribution of anteroposterior (AP) contrast spread grades differed significantly between the two trajectory groups (Figure 4, Table 2, *p* < 0.001).

In the inward trajectory group, most patients exhibited Grade 1 spread, whereas Grade 2 spread was relatively uncommon. In contrast, the outward trajectory group showed a predominance of Grade 2 spread, with no cases of Grade 0 spread.

**Figure 5:**
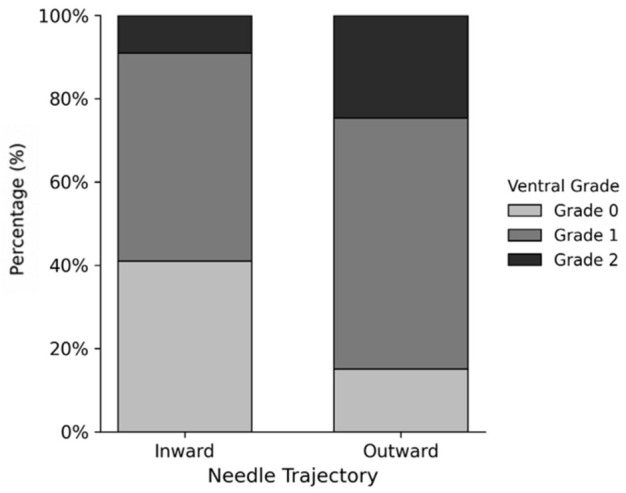
Distribution of ventral epidural contrast spread grades according to needle trajectory.

A similar trend was observed for ventral epidural spread, with the outward trajectory group demonstrating higher ventral grades compared to the inward group (Figure 5, *p* = 0.004).

### 3.3. Association Between Needle Trajectory and Pain Reduction

**Figure 6:**
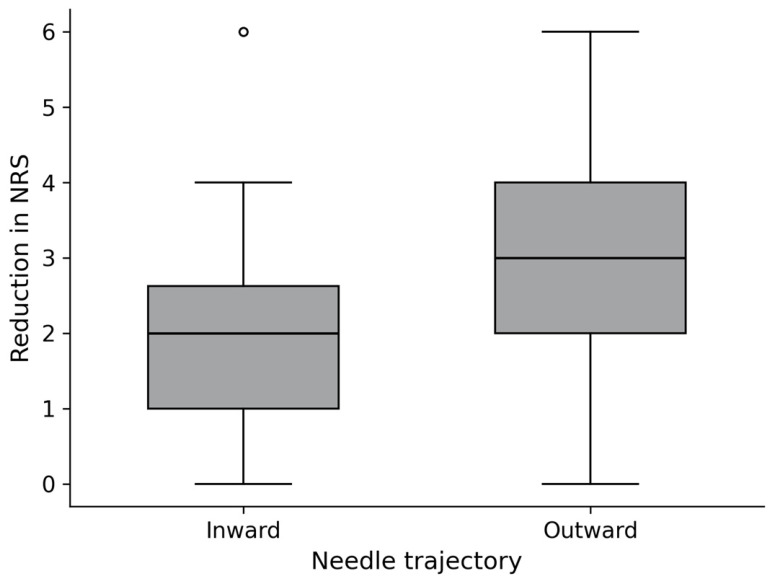
Comparison of pain reduction according to needle trajectory. Circles indicate outliers. NRS: numeric rating scale.

Pain reduction was significantly greater in the outward trajectory group than in the inward trajectory group, with a mean difference of 1.25 (95% CI: 0.74–1.76, *p* < 0.001) and a large effect size (Cohen’s d = 0.96, 95% CI: 0.56–1.36) (Figure 6).

### 3.4. Association Between AP Contrast Spread Pattern and Pain Reduction

**Figure 7:**
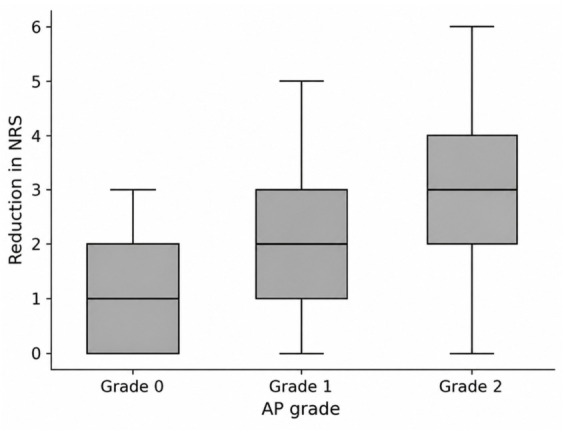
Association between pain reduction and AP contrast spread grade. NRS: numeric rating scale.

Higher AP contrast spread grades were significantly associated with greater pain reduction (β = 0.83, 95% CI: 0.44–1.22, *p* < 0.001), with a moderate-to-large effect size (η^2^ = 0.14). The relationship between AP contrast spread grade and pain reduction is illustrated in Figure 7.

### 3.5. Multivariable Analysis

In multivariable linear regression analysis adjusting for age, sex, baseline NRS, and target level, needle trajectory remained significantly associated with pain reduction (β = 0.98, 95% CI: 0.36–1.61, *p* = 0.002), whereas AP contrast spread grade was not independently associated with pain reduction (Table 3).

### 3.6. Association Between Ventral Epidural Spread and Pain Reduction

Although ventral epidural spread showed a trend toward greater pain reduction, the association did not reach statistical significance (Kruskal–Wallis test, *p* = 0.081).

## 4. Discussion

The present study demonstrates that needle trajectory within the paramedian cervical interlaminar approach is associated with contrast distribution patterns and short-term pain reduction. Specifically, an outward-directed trajectory resulted in a higher proportion of foraminal or periradicular contrast spread and greater pain relief. Given the observational nature of this study, these findings should be interpreted as associations rather than causal relationships.

Previous studies have shown that epidural injectate spread is highly variable and influenced by anatomical constraints and procedural technique [16,17]. In particular, conventional interlaminar injections may not reliably deliver medication to the neural foramen or periradicular region, which may contribute to variable clinical outcomes [15,18,22]. Although contrast distribution has been suggested as a surrogate marker for therapeutic effectiveness, it does not necessarily reflect actual drug delivery or predict analgesic efficacy [16,17].

In this context, the present study suggests that needle trajectory may influence contrast distribution patterns and, consequently, clinical outcomes. While prior studies have reported inconsistent relationships between imaging findings and pain relief, we observed that trajectory-related differences in contrast distribution were associated with measurable differences in pain reduction. However, this relationship was not maintained after adjustment for confounding variables, indicating that the findings should be interpreted cautiously.

Because needle trajectory likely directly influences AP contrast spread, a potential mediation or collinearity relationship between these variables should be considered when interpreting the multivariable model. The loss of independent statistical significance of AP contrast spread after adjustment may therefore reflect shared variance between trajectory and contrast distribution patterns rather than absence of clinical relevance.

Notably, a moderate-to-large effect size was observed in univariable analysis, suggesting that the magnitude of the association may still be clinically relevant despite the lack of independent association [23].

A plausible explanation is that an outward-directed trajectory may facilitate contrast extension toward the foraminal or periradicular region, potentially improving drug delivery to the affected nerve root [15,18,24]. Nevertheless, this interpretation remains speculative and should be interpreted with caution.

Notably, ventral epidural spread was not significantly associated with pain reduction, suggesting that lateral or foraminal distribution may be more clinically relevant than anterior spread in this context.

From a clinical perspective, these findings suggest that careful attention to needle trajectory may enhance both contrast distribution and short-term analgesic outcomes. Importantly, the observed reduction in NRS scores suggests that the findings may be clinically meaningful, although this should be interpreted with caution and in the context of established thresholds for clinical relevance. However, the magnitude and durability of this effect remain to be established.

From a safety perspective, altering needle trajectory toward a more lateral direction may theoretically increase the risk of unintended needle placement or proximity to critical neurovascular structures [10,11,13,25]. Therefore, careful fluoroscopic guidance and detailed anatomical knowledge are essential when applying this technique.

In addition, alternative explanations for the observed pain reduction, such as placebo effects, natural disease progression, or regression to the mean, cannot be excluded and should be considered when interpreting the results.

Several limitations should be acknowledged. First, the retrospective design introduces the possibility of selection bias and limits causal inference. Therefore, the observed associations should be interpreted with caution, as unmeasured confounding factors may have influenced the results. Second, outcomes were assessed only at 2 weeks, and the long-term clinical significance of the findings remains uncertain. Third, contrast grading was based on a semi-quantitative fluoroscopic assessment and may be subject to observer-related variability despite high inter-rater agreement. Fourth, functional outcomes and quality-of-life measures were not evaluated. Although pain reduction is a clinically relevant outcome, it may not fully capture the overall impact of treatment. Fifth, potential confounding factors may not have been fully accounted for despite multivariable adjustment. Finally, the absence of a direct comparison with other approaches limits the generalizability of our findings.

All procedures were performed by a single experienced operator, which ensured technical consistency but may limit the generalizability of the findings. In addition, techniques requiring precise control of needle trajectory may be influenced by operator experience and familiarity with fluoroscopic guidance, suggesting a potential learning curve. Therefore, the reproducibility of these findings in less experienced settings remains uncertain.

Future studies incorporating advanced imaging modalities, such as computed tomography, magnetic resonance imaging, or virtual reality-based anatomical simulation, may allow more precise evaluation of needle trajectory and injectate distribution, thereby improving the anatomical validation of trajectory-based techniques and providing further insight into their mechanisms [26].

Because the contrast volume was generally limited to a relatively narrow range (1–2 mL), major differences in contrast spread patterns are less likely to be explained solely by volume variability. However, the potential influence of contrast volume cannot be completely excluded.

Despite these limitations, the present study provides evidence suggesting that needle trajectory may represent a clinically relevant technical factor influencing both contrast distribution patterns and clinical outcomes in paramedian cervical interlaminar epidural steroid injection, although these findings should be interpreted within the context of the study design and analytical limitations.

Future prospective studies with standardized protocols and longer follow-up are warranted to confirm these findings and to determine their long-term clinical significance.

## 5. Conclusions

Needle trajectory within the paramedian cervical interlaminar approach was associated with contrast distribution patterns and short-term pain reduction. An outward-directed trajectory was associated with greater foraminal or periradicular contrast spread and greater pain reduction.

Although contrast spread patterns were associated with pain reduction in univariable analysis, they were not independently associated after adjustment for confounding variables. These findings suggest that needle trajectory may represent an important procedural factor influencing clinical outcomes.

These findings support the potential clinical relevance of needle trajectory as a procedural factor in paramedian cervical interlaminar epidural steroid injection.

## Figures and Tables

**Table 1:** Baseline characteristics of the study population.

Variable	Total (n = 109)	Inward (n = 56)	Outward (n = 53)	*p*-Value
Age (years)	55.8 ± 11.3	56.6 ± 11.8	55.0 ± 10.8	0.460
Sex (M/F)	63/46	32/24	31/22	0.880
Level (%)				0.980
C4–5	24 (22.0%)	13 (23.2%)	11 (20.8%)	
C5–6	52 (47.7%)	27 (48.2%)	25 (47.2%)	
C6–7	25 (22.9%)	12 (21.4%)	13 (24.5%)	
C7–T1	8 (7.3%)	4 (7.1%)	4 (7.5%)	
Side (R/L)	43/66	23/33	20/33	0.840
Baseline NRS	5.4 ± 1.6	5.2 ± 1.5	5.6 ± 1.7	0.180
2-week NRS	2.7 ± 1.8	3.5 ± 1.7	1.9 ± 1.5	<0.001
Pain reduction	2.3 ± 1.8	1.71 ± 1.4	2.96 ± 1.8	<0.001

Data are presented as mean ± standard deviation or number (%). NRS, numeric rating scale. Cohen’s d for pain reduction = 0.96 (large effect).

**Table 2:** Distribution of anteroposterior (AP) contrast spread according to needle trajectory.

AP Grade	Inward (n = 56)	Outward (n = 53)	Total (n = 109)
Grade 0	11 (19.6%)	0 (0.0%)	11 (10.1%)
Grade 1	40 (71.4%)	16 (30.2%)	56 (51.4%)
Grade 2	5 (8.9%)	37 (69.8%)	42 (38.5%)

*p* < 0.001 (chi-square test).

**Table 3:** Multivariable linear regression analysis for factors associated with pain reduction.

Variable	β Coefficient	95% CI	*p*-Value
Trajectory (Outward vs. Inward)	0.98	0.36 to 1.61	0.002
AP grade 1 (vs. Grade 0)	0.35	−0.51 to 1.21	0.427
AP grade 2 (vs. Grade 0)	0.48	−0.55 to 1.50	0.365
Age	−0.014	−0.037 to 0.009	0.234
Sex (Male vs. Female)	−0.19	−0.69 to 0.32	0.471
Baseline NRS	0.29	0.14 to 0.43	<0.001
Level (C5–6 vs. C4–5)	−0.33	−0.89 to 0.23	0.249
Level (C6–7 vs. C4–5)	−0.06	−0.76 to 0.64	0.867
Level (C7–T1 vs. C4–5)	−0.13	−1.24 to 0.97	0.810

Values are presented as β coefficients with 95% confidence intervals. NRS, numeric rating scale.

## Data Availability

The data presented in this study are available on request from the corresponding author. The data are not publicly available due to privacy restrictions.

## Abbreviations

The following abbreviations are used in this manuscript:

CESI	Cervical interlaminar Epidural Steroid Injection
TFESI	TransForaminal Epidural Steroid Injection
AP	AnteroPosterior
CLO	ContraLateral Oblique
